# Medical device procurement in low- and middle-income settings: protocol for a systematic review

**DOI:** 10.1186/2046-4053-3-118

**Published:** 2014-10-21

**Authors:** Karin Diaconu, Yen-Fu Chen, Semira Manaseki-Holland, Carole Cummins, Richard Lilford

**Affiliations:** 1 Public Health, Epidemiology and Statistics Unit, Department of Health and Population Sciences, University of Birmingham, B15 2TT Edgbaston, West Midlands, UK; 2 Health Sciences, University of Warwick, Room 155, Coventry CV4 7AL, UK

**Keywords:** Developing countries, Prioritization, Procurement, Medical devices

## Abstract

**Background:**

Medical device procurement processes for low- and middle-income countries (LMICs) are a poorly understood and researched topic. To support LMIC policy formulation in this area, international public health organizations and research institutions issue a large body of predominantly grey literature including guidelines, manuals and recommendations. We propose to undertake a systematic review to identify and explore the medical device procurement methodologies suggested within this and further literature. Procurement facilitators and barriers will be identified, and methodologies for medical device prioritization under resource constraints will be discussed.

**Methods/design:**

Searches of both bibliographic and grey literature will be conducted to identify documents relating to the procurement of medical devices in LMICs. Data will be extracted according to protocol on a number of pre-specified issues and variables. First, data relating to the specific settings described within the literature will be noted. Second, information relating to medical device procurement methodologies will be extracted, including prioritization of procurement under resource constraints, the use of evidence (e.g. cost-effectiveness evaluations, burden of disease data) as well as stakeholders participating in procurement processes. Information relating to prioritization methodologies will be extracted in the form of quotes or keywords, and analysis will include qualitative meta-summary. Narrative synthesis will be employed to analyse data otherwise extracted. The PRISMA guidelines for reporting will be followed.

**Discussion:**

The current review will identify recommended medical device procurement methodologies for LMICs. Prioritization methods for medical device acquisition will be explored. Relevant stakeholders, facilitators and barriers will be discussed. The review is aimed at both LMIC decision makers and the international research community and hopes to offer a first holistic conceptualization of this topic.

## Background

Medical devices and equipment are crucial for quality health service delivery. Reports and research on low- and middle-income countries cite a lack of basic medical devices as well as medical equipment falling into disuse within these settings [1,2]. This severely impairs health care provision and also translates to lost resources and funds. The WHO Priority Medical Devices project suggests two potential causes for this problem [2]. First, medical device manufacturers target high-income country economies due to a higher potential profit. Thus, medical device supply and equipment design are restricted to products and specifications suitable for deployment settings with advanced infrastructure and technologically knowledgeable human resources. Second, issues around the judicious procurement of medical devices arise for low- and middle-income countries (LMICs) [see Additional file 1: Definitions: Medical device procurement]. Inappropriate selection of products impedes equipment uptake and use within deployment settings. Medical devices should be appropriate for and readily available in LMIC settings as well as accessible and affordable for health care facilities, their staff and national governments [2-5].

However, little is known about how medical device procurement does or should take place within LMICs, and processes may substantially differ from those employed in high-income countries (HICs). Within the latter settings, technology acquisitioning processes are guided by principles of quality care delivery and value for money to ensure containment of rising health care costs. A diverse range of stakeholders is involved in deliberation of potential purchases: clinicians, public health commissioners, researchers and patients. The review of clinical and cost-effectiveness evidence as well as value-based criteria such as equity form the basis of such deliberations [6-10]. The WHO Baseline Country Survey on medical devices illustrates that in contrast to HICs, LMICs undertake medical device procurement at national rather than regional or facility level ([11] and Table 1: Author’s calculation: chi-square with 3 degrees of freedom, total sample *n* =162, *p* <0.01). The survey fails, however, to provide more granular detail on stakeholders involved in these processes as well as principles pursued—e.g. is cost-effectiveness a desired feature of potential purchases?

**Table 1:** Procurement of medical devices at national level in relation to country income classification (World Bank 2014)

**Country classification**	**Does procurement of medical devices occur at national level?**	
	**(Responses from WHO Baseline Country Survey 2010)**	
	**Yes**	**No**
Low income	25	8
Low-middle income	31	7
Upper-middle income	30	17
High income	17	27
Total	103	59

Author’s calculation: chi-square with 3 degrees of freedom, total sample *n* =162, *p* <0.001.

To guide decision makers in the procurement of medical devices for LMICs, numerous recommendations, guidelines and tools have been issued by international think tanks and public health organizations. Substantial heterogeneity can be observed in relation to these: recommendations may focus on procurement for specific interventions or service delivery packages, clinical areas or specialties, as well as entire health facilities and ancillary services offered [12-14]. The WHO itself recommends medical equipment selection for procurement take the shape of ‘availability matrices’ [15]. This involves targeting clinical areas and interventions associated to a country’s highest burden of disease and identifying medical equipment key for investment in or provision of said services.

To date, no systematic review and appraisal of the literature around medical device procurement recommendations, guidelines and research exists. We propose to undertake such a systematic review to identify how medical device procurement and prioritization within LMICs should take place in the future, based on research which reports on procurement and prioritization processes as well as recommendations put forth in publicized guidelines and similar materials. The current paper serves as a study protocol for this exercise. We believe that a systematic review on this topic would prove beneficial to decision makers and procurement practitioners within LMICs by helping identify initial core principles for equipment purchasing. Further, we wish to explore prioritization methodologies proposed within the literature. Under resource constraints, prioritization is a crucial part of a procurement process and directly informs equipment selection. Medical device-specific prioritization criteria will be identified, and this may inform the wider debate on how prioritization processes are shaped and implemented [16-18]. Identified principles and methodologies will be discussed and interpreted in light of information relating to settings described, type of medical equipment proposed for procurement, as well as type of issuing organization and reason for document development.

The main research question is: What methods inform or are recommended for LMIC specific medical device and equipment procurement? In the course of exploring the above study question, we also expect to consider: the evidence base used to inform medical device procurement methods and processes and the factors impacting upon medical device procurement and the methods proposed for medical device prioritization.

## Methods/design

### Search strategy

Early scoping searches on medical device procurement methods for LMICs revealed a large body of grey literature, issued by international public health agencies, think tanks or similar institutions, but very few journal articles or research studies. It was therefore important to design search and selection strategies to be as broad and inclusive as possible, with no time or language restrictions. The range of documents to be included will, however, be restricted to freely available digitized material, partly due to resource constraints, partly because we believe this most closely mirrors the various materials that LMICs would be able to access. We acknowledge this as a limitation of the study; however, scoping searches indicate that the majority of documents to be retrieved are part of the grey literature and digitized and freely available through the World Health Organization and ancillary institutions. A full list of sources to be searched is provided in Table 2.

**Table 2:** Type of search conducted and sources searched

**Search type**	**Search sources**	
OVID MEDLINE search algorithm and keyword searches	Bibliographic databases	OVID MEDLINE, OVID Embase, Cochrane Library, CRD databases (DARE, NHS EED, HTA), CEA Registry, LILACS, African Index Medicus, Econlit, HMIC
Keyword searches	Website searches	TRIP, National Guideline Clearinghouse, International Guideline Library, NHS Evidence and Clinical Evidence (NICE), Clinical Evidence (BMJ), INAHTA, CADTH, HTAi, Web of Science, CHE York, CHEPA, Cost Effectiveness Analysis Registry, Office of Health Economics
	Organizational databases/websites	WHO Health Technology e-documentation centre, WHO, UNDP, UNICEF, UNAIDS, WB Group (IBRD particularly), MSF, AfDB, ADB, EBRD
	National donor agencies	DFID, USAID (including MSH), AUSAID, GIZ, BMZ, JICA, and other relevant agencies that may be identified during the search
	Grey literature databases	ZETOC, CPCI
Contacting experts	Contact with experts to identify additionally relevant literature	

To identify relevant documents from the literature, search terms grouped around three distinct topics will be employed: medical devices and equipment, procurement and LMICs. Guided by a consensus definition of medical devices [19], the review will focus on any type of medical device ranging from consumables (e.g. bandages, needles) to routine medical equipment (e.g. stethoscopes, ECG machines) and devices (e.g. condoms) as well as medical furniture (e.g. delivery beds). Search terms will refer to medical devices and equipment, medical supplies and medical or biomedical technologies and will include relevant subject headings. Further search terms and subject headings include synonyms for procurement and terms around LMICs and income levels.

An OVID MEDLINE search string is provided in Table 3. Where possible, keyword combinations similar to the search string provided will be used in all sources in order to identify the relevant material. No restrictions around the specific type of material to be retrieved will be employed: databases, reports, notices, presentations, conference proceedings, journal articles, manuals and books will all be reviewed provided that they are freely available and digitized. Native language speakers will be identified to assess, select and report on non-English studies, thus, limiting potential translation bias.

**Table 3:** Example of search strategy for MEDLINE (OVID SP) up to week 2 of January 2013

**No.**	**Search strategy**
1.	device.mp. or exp “Equipment and Supplies”/
2.	(device* or equipment* or suppl*).mp. [mp = title, abstract, original title, name of substance word, subject heading word, keyword heading word, protocol supplementary concept, rare disease supplementary concept, unique identifier]
3.	exp Technology, Radiologic/ or exp Technology Assessment, Biomedical/ or exp Fiber Optic Technology/ or exp Educational Technology/ or exp Biomedical Technology/ or technology.mp. or exp “United States Office of Technology Assessment”/ or exp Technology/ or exp Food Technology/ or exp Technology, High-Cost/ or exp Technology Transfer/ or exp “National Center for Health Care Technology (U.S.)”/ or exp Wireless Technology/ or exp Technology, Dental/ or exp Green Chemistry Technology/ or exp Technology, Pharmaceutical/ or exp Remote Sensing Technology/
4.	1 or 2 or 3
5.	(procure* or purchas* or acqui* or commission* or buy*or order*).mp. [mp = title, abstract, original title, name of substance word, subject heading word, keyword heading word, protocol supplementary concept, rare disease supplementary concept, unique identifier]
6.	(countr* adj2 (income or poor or poverty or develop* or resource or low* or mid*)).mp. [mp = title, abstract, original title, name of substance word, subject heading word, keyword heading word, protocol supplementary concept, rare disease supplementary concept, unique identifier]
7.	(third adj2 world).mp. [mp = title, abstract, original title, name of substance word, subject heading word, keyword heading word, protocol supplementary concept, rare disease supplementary concept, unique identifier]
8.	(emerging adj2 (econom* or market*)).mp. [mp = title, abstract, original title, name of substance word, subject heading word, keyword heading word, protocol supplementary concept, rare disease supplementary concept, unique identifier]
9.	developing country.mp. or exp Developing Countries/
10.	6 or 7 or 8 or 9
11.	4 and 5 and 10

Conducted last: 28 January 2013, 15:10 (GMT). Number of records identified: 2,297.

### Selection and inclusion

**Figure 1:**
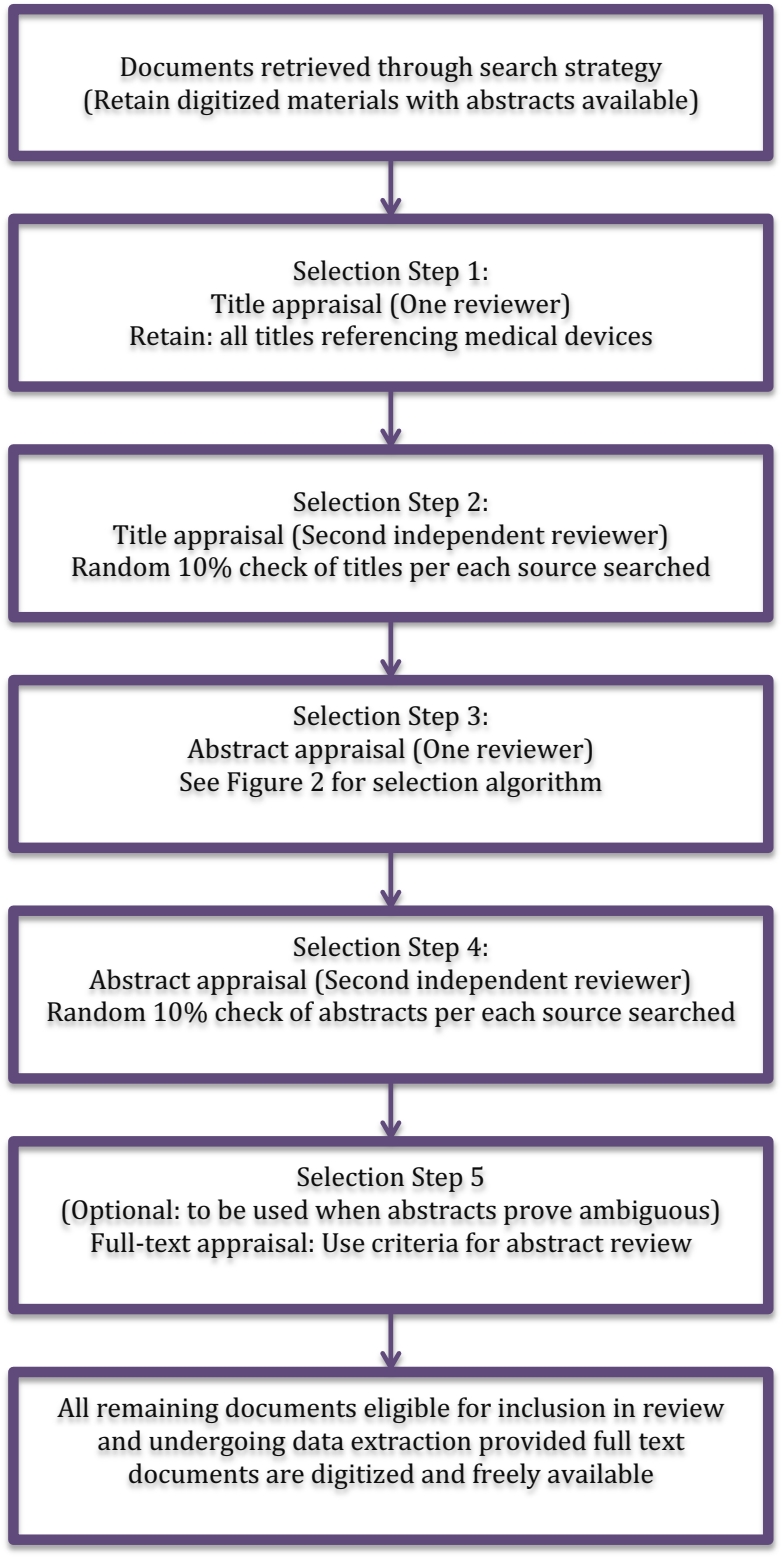
**Study selection algorithm.** Method for study selection to be employed by the reviewers.

All records identified in the search will then be screened for potential inclusion into the review (see Figure 1 for a selection algorithm). At first stage, only titles will be considered and all documents mentioning medical devices, either specific devices or equipment/supplies in general or interventions likely to make use of equipment (e.g. vaccinations, orthopaedic surgery) will be retained. This is to ensure that documents are indeed focused on the topic of interest. One researcher will undertake title review; however, a second independent researcher will check a random 10% sample of documents for each of the sources searched. Any disagreements will be resolved through discussion or consultation with a third reviewer.

**Figure 2:**
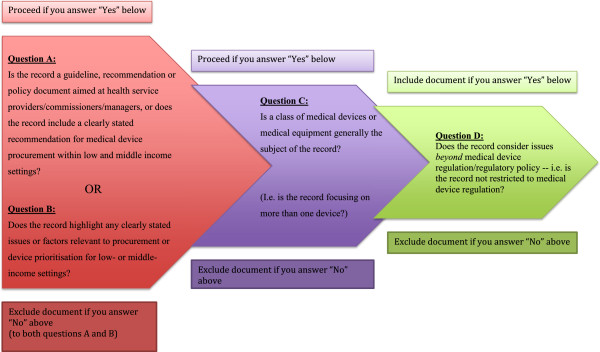
**Abstract and full-text inclusion and exclusion criteria.** Method for abstract selection to be employed by the reviewers.

Abstracts will then be reviewed in light of four pre-specified selection criteria or questions (Figure 2). These are directly linked to the outcome questions to be investigated and are formulated so as to retain documents including recommendations or discussions of medical device procurement and prioritization processes, or documents clearly indicating factors which may impact upon medical device procurement. In addition, we have chosen to include only documents discussing processes relating to the procurement of more than one device: this is because we consider that any prioritization process potentially employed in procurement would fundamentally rely on the comparative assessment or evaluation of more than one technology/product. Reviewers will, however, take into account that documents may restrict their focus to one device while still including a discussion on the relative merits of similar devices: e.g. a document on the procurement and pre-qualification of a particular intrauterine contraceptive device may still be included provided that it includes a more detailed discussion on similar devices and their specifications [20]. Documents discussing only regulatory issues relating to procurement or medical device supply have also been excluded as they are considered too narrow in focus to provide meaningful information on the outcome question set.

The selection questions to be used are detailed below and have been piloted by two independent reviewers on a sample of 20 documents retrieved from OVID MEDLINE and the WHO E-Health Technology Documentation Centre. The latter database has been identified in scoping searches as including a large body of relevant material and was therefore considered suitable for piloting. The questions were deemed appropriate for the purposes of this study and are outlined below along with examples of documents identified in the piloting process as appropriate/inappropriate for inclusion. These examples were made available to reviewers for consultation during the study selection phase of the review.

A. Is the record a guideline, recommendation or policy document aimed at health service providers/commissioners/managers, or does the record include a clearly stated recommendation for medical device procurement within low and middle income settings?

Example of ‘yes’: World Health Organization [21]: Procuring Single-use Injection Equipment and Safety Boxes. The executive summary indicates that the objective of the document is to “accompany pharmacists, physicians, procurement staff and programme managers through the process of procuring single-use injection equipment and safety boxes of assured quality, on a national or international market, at reasonable prices”.

Example of ‘no’: Ross et al. [22]. Study protocol: Ethics, Economics and the Regulation and Adoption of New Medical Devices: Case Studies in Pelvic Floor Surgery [22]. Rejected because the methods section of the abstract indicates this study uses examples from a Canadian context; no link to LMICs is stated.

B. Does the record highlight any clearly stated issues or factors relevant to procurement or device prioritisation for low- or middle-income settings?

Example of ‘yes’: Anderson, B. O et al. [23]. Optimisation of breast cancer management in low-resource and middle-resource countries: executive summary of the Breast Health Global Initiative consensus, 2010 [23]. Abstract indicates that journal article includes a discussion on “programme infrastructure and capacity (including appropriate equipment and drug acquisitions, and professional training and accreditation).”

Example of ‘no’: Porto, J. P et al. [24]. Nosocomial infections in a paediatric intensive care unit of a developing country: NHSN 45(4), 475–479 [24]. Rejected because abstract does not mention procurement or device prioritization.

C. Is a class of medical devices or medical equipment generally the subject of the record? (i.e. is the record focusing on more than one device?)

Example of ‘yes’: Kalifa et al. [25]. Imaging in pediatrics. Strategy and economic implications for the Third World, Annales de Pediatrie 39(2): 67–70 [French] [25]. Abstract mentions medical imaging equipment and provides two distinct examples: ultrasonography and roentgenography.

Example of ‘no’: Malkin, R., Anand, V. [26]. A Novel Phototherapy Device ©. [26]. Rejected because abstract indicates document focuses on product development of a single device.

D. Does the record consider issues *beyond* medical device regulation/regulatory policy—i.e. is the record not restricted to medical device regulation?

Example of ‘yes’: Kalifa et al. [25]. Imaging in pediatrics. Strategy and economic implications for the Third World, Annales de Pediatrie 39(2): 67–70 [French] [25]. Abstract indicates that document content is not restricted to device regulation, instead focusing on two medical device classes and their use in LMICs.

Example of ‘no’: World Health Organization [27]. Medical Device Regulations: Global Overview and Guiding Principles [27]. Rejected because document is restricted to a discussion on global regulatory frameworks and principles.Please note that the selection questions are used as detailed in Figure 2. This means that documents will be included in the review if answers:

● A, C, D = Yes

● B, C, D = Yes

● A, B, C, D = Yes

The selection criteria will be re-evaluated as necessary by reviewers, and any amendments to this original study protocol will be noted in the published systematic review. We acknowledge that abstracts may prove ambiguous, and that reviewers may therefore wish to refer to full-text documents at times. When this is needed, reviewers should make use of the same four questions specified above for study selection, and note that, a full-text review has been carried out. A random 10% sample of abstracts obtained from each of the sources searched will undergo screening by a second independent reviewer, and all disagreements will be resolved through discussion or consultation with a third reviewer.

### Data extraction

All documents which were screened and deemed eligible will be included in the proposed study. The task of data extraction will be split across reviewers, who will read full-text documents to obtain data on a pre-specified list of variables and questions (see Table 4: Data extraction template). Similar to the selection criteria, the data extraction template has been piloted on a random sample of 17 documents which were deemed eligible for inclusion from OVID MEDLINE and the WHO E-Health Technology Documentation Centre.

**Table 4:** Data extraction template

**No.**	**Question/item**	**Tick if applicable**	**Answer (if applicable)**
			**Example answers below**
1.	Study ID + bibliographic information		
2.	Type of record		
	● Is the record a guideline/recommendation/policy document or an academic article?		
	● Is the document part of a greater study/document? (if so, appraise that document as well but link information relating to evidence)		
	● Are the authors contactable?		
3.	Institution of origin and who the institution reports to		
	● Record institution (if this is an academic article, record university)		
	● Why did the institution develop the record?		
	● Under what remit does the institution operate? (e.g. if university was commissioned to develop record, record how the institution commissioning the research will use the record, if specified)		
4.	Setting/country of origin and any information regarding the below (note if specified in record)		
	● Economic and development indicators: HDI level, GDP, GDP/capita, Health expenditure as % GDP, % total government expenditure or medical device expenditure as % of health budget		
	● What does the disease burden look like? Is any epidemiological evidence presented?		
	● How is health care funded?		
	● What other factors related to country/countries in question are mentioned (e.g. income inequality, access to health care, national security, infrastructure, health service infrastructure)?		
5.	Methodological data: Answers to be recorded to the below questions from the record considered.		
	1) Is prioritization of medical device purchasing/selection explicit?		
	a) If yes, describe the method presented in the record and further evaluate below questions.		
	2) Is it clear who/what institutions hold the responsibility for medical device management?		
	a) If yes, note the institutions and their remit (e.g. national, international).		
	3) Are budget impact (national, local or by facility level) or national health care/service funding policies mentioned and if so is any relation to procurement or prioritisation made explicit?		
	4) Is health technology assessment mentioned?		
	Health technology assessment example phrases: evidence base; clinical and cost effectiveness data; HTA appraisal systems; HTA process—i.e. timing, cost, staffing, expertise, national/international remit.		
	a) If yes, is it clear how the HTA evidence is integrated into the prioritisation and procurement decisions? Describe the mechanism.		
	b) Is it clear who is responsible for HTA appraisal and for issuing recommendations? Who has access to the HTA evidence? How is this disseminated?		
	5) Are direct care providers mentioned?		
	Examples of direct care providers: nurses, medics, volunteers etc.		
	a) If so, is it clear what their influence on purchasing/prioritisation is? (e.g. do they directly commission, do they prefer certain suppliers)		
	b) Are any issues regarding staff training and ability to deliver services mentioned? (e.g. staff may not be trained to use a particular device)		
	c) Is it clear how staff is involved in the maintenance of medical devices?		
	6) Are care pathways or clinical guidelines mentioned?		
	Examples of clinical guidelines: WHO guidelines for diabetes management, etc.		
	a) Is it clear what clinical guidelines or care pathway information was used in device selection or prioritisation?		
	7) Is health needs assessment mentioned?		
	a) If yes, what are the health priorities of the population in question and how were they derived in the HNA process?		
	b) Is it clear how the health needs assessment informed procurement decisions?		
	8) Are commissioning strategies for health services and equipment mentioned?		
	For example: Afghanistan’s MSH guide on “Equipment for BPHS for Health Posts” refers to a national procurement strategy so both documents would need to be evaluated and the national procurement strategy would form the basis for the guide assessed.		
	a) If yes, record what types of strategies are mentioned? (e.g. national, international)		
	b) If yes, what is the relation of said strategy to the current record being assessed? Does one form the basis of the other, do they operate complementarily and is adherence to one or the other or both mandatory?		
	c) Follow up on the national or local strategy and undertake a record assessment.		
	9) Are health service delivery facilities (e.g. hospitals, health centres, mobile units) mentioned?		
	a) If yes, which facilities are directly mentioned?		
	b) If yes, is it clear which medical devices are a priority for each facility or facility level?		
	c) If yes, are ambient conditions of the facility necessary for device performance mentioned? (e.g. running water, electricity availability)		
	d) If yes, and if a device list is present, is it clear if device purchasing was restricted to a particular class of devices: e.g. consumables that do not require electricity, mobile devices that need little maintenance, etc.		
	10) Does the record mention expert advice on the device procurement/prioritisation?		
	a) If yes, what kind of expert would be consulted and what documentation do said experts provide to inform device procurement/prioritisation?		
	11) Are particular standards regarding devices mentioned? Mentions of standardization regarding devices could include naming specific brands, product specifications, specific suppliers, specific regulatory nomenclatures)		
	12) Are any costs mentioned in the record?		
	a) If so, record which costs are mentioned.		
	13) Are execution strategies mentioned in regards to particular health campaigns? (either of national or international importance)		
	Examples include: HIV/AIDS campaigns which are commissioned through UNAIDS		
	a) If yes, who/what institution advises on device procurement and prioritisation?		
	b) Is it clear what the recommendations regarding device procurement are? Note down recommendations.		
	14) Are more up to date versions of lists/guidelines/methods of the same record present?		
	a) If yes, appraise newer record versions as well.		
	15) Is evidence of evaluation strategies regarding procurement lists, guides, methods present?		
	a) What evaluation strategies are mentioned?		
	b) Who undertakes said evaluations?		
	c) Is it clear what evidence is being used to inform evaluations?		
6.	Equipment related data: Answers to be recorded to the below questions.		
	1) What are the main categories of equipment included in record?		
	a) Renewable supplies		
	b) Surgical supplies		
	c) Condition specific		
	d) Record the equipment categories mentioned.		
	2) How detailed is the equipment specification? (i.e. are measurements mentioned; is a description provided;)		
	3) How many distinct products are mentioned?		
	4) Is a mix of devices mentioned and is it clear if certain devices are complementary (i.e. they need to be used in conjunction with one another)?		
	5) Does the list mention how many items of one product to purchase?		
	6) Are any national/regional device coverage targets set? (i.e. how many devices/institution/region		
	7) Is any cost data present and if so, note down what cost data is available.		
	8) Is any information on installation available and if so, note what recommendations are given.		
	9) Is any information on maintenance available and if so, note what recommendations are given.		
	10) Is any information on necessary ambient conditions supplied and if so, note what said conditions are. (i.e. “needs constant electricity supply”)		
	11). Is any recommendation regarding device disposal given and if so note what said recommendation is.		
7.	Capacity building: Answers to be recorded to the below questions.		
	1) Does the record outline any strategies for training people in medical device purchasing or medical device management?		
	a. If yes, record what said strategies are.		
8.	Notes		
	Recording of any additional information that seems of relevance.		
	Example: WHO Priority Medical Devices frequently refers to diagnostic coding systems and disability classification systems.		

Provided as Excel spreadsheet to reviewers. Data extraction template provided to reviewers.

To address our study aims and outcome questions, data relating to the following five domains will be extracted:

● Document identifier and characteristics: This covers information unique to the document (e.g. authors, year of publication, journal) as well as a categorization of the document according to purpose of publication (e.g. guideline, research study).

● Described setting: Information on country descriptions will be noted where available in order to provide a context to data extracted and further interpretation.

● Methodological data: This is the most substantial task and covers information relating to prioritization and procurement methodologies as well as factors affecting procurement processes. Where explicit prioritization methodologies are described, reviewers will be instructed to extract quotes or keywords describing these processes in order to allow for close textual interpretation. Further questions require reviewers to provide dichotomous ‘yes/no’ answers relating to the use of evidence in procurement (e.g. use of cost-effectiveness evidence, health needs assessments), availability of procurement policies/frameworks (e.g. health technology management frameworks), influence of stakeholders (e.g. which institutions or facilities affect the process of procurement) as well as influence of processes/health campaigns (e.g. quality assurance, targeted programmes or interventions). For any additional information, reviewers wish to capture, an additional “notes” section is provided.

● Equipment related data: Any information available related to the equipment to be procured is captured here: clinical area equipment is used in, equipment specification, cost of procurement, and maintenance, installation or decommissioning information among others.

● Capacity building: Reviewers are asked to note any proposed training strategies related to medical device procurement in LMICs.

### Analysis and interpretation

Two methods of data analysis will be employed for this systematic review, each corresponding to the type of data extracted. Where reviewers are tasked with extracting quotes or keywords, relating in particular to prioritization methods described in the literature, qualitative meta-summary was deemed the most appropriate method for analysis [28]. Treating extracted quotes and keywords as a primary (i.e. uninterpreted) description of phenomena that document authors wish to report, qualitative meta-summary proposes the grouping of topically similar data and the generation of further abstractions aimed at describing underlying themes and processes. This allows for a richer contextual interpretation of data, something particularly valuable when trying to generate initial theses in relation to how medical device procurement and prioritization is viewed within the literature.

For data otherwise extracted, i.e. dichotomous data extracted on remaining pre-specified variables, narrative synthesis was deemed appropriate [29]. In the first instance, this will entail generating descriptive statistics and examining associations between variables through the use of chi-square (or Fisher’s exact) tests as appropriate. Associations between the following variables may be investigated: presence of health technology management frameworks (and actors engaged in technology management) and use of commissioning strategies for procurement, health service delivery levels, evidence in procurement (e.g. health needs assessments) as well as health facility equipment priorities and assigned maintenance responsibilities for health care facility staff. Further explorations will focus on the disease areas or type of equipment cited and specifications recorded for these in addition to instructions on deployment in health facilities and human resource training levels, as well as installation and maintenance necessities. The influence of publication year issuing organization and reason for document development on details associated to the above variables may also be explored. Documents may also be grouped according to their type (e.g. research studies, guidelines) to highlight potential differences in reporting on procurement or prioritization processes. Capacity building strategies related to procurement will be discussed. Mind-maps showcasing associations may be created to provide visual representations.

### Reporting

For reporting purposes, we will follow the PRISMA statement for systematic reviews and refer readers to this protocol for further clarifications [30]. We expect that we will not be able to report on all items in the statement, e.g. relating to risk of bias within or across studies (items 12, 15, 19, 22) or to quantitative outcomes, synthesis of results or additional subgroup analyses (items 13, 20, 21). Outcomes will be discussed as aforementioned through the use of qualitative meta-summary or narrative synthesis. Registration with PROSPERO is not appropriate in the case of this review, as it does not concern itself with a clinical intervention.

## Discussion

It is unclear how medical device procurement and prioritization take place within LMICs. Internationally proposed guidelines, recommendations or reports—whether developed by public health agencies or research institutions—are routinely issued to counsel LMICs on this topic and may impact upon their national policy formulation. It is therefore germane to understand the procurement/prioritization methodologies proposed within this literature. The aims of this systematic review are to identify said methodologies, explore the factors reported as affecting procurement practices in LMICs and create an initial framework for how medical device prioritization and procurement should be designed and conceived in resource-constrained settings.

We acknowledge several limitations of the proposed endeavour. First, we note the difficulty associated with undertaking a first-line review on a topic associated with methodologically diverse literature. We expect that documents reviewed will range from procurement notices and emergency medical device lists to procurement manuals or research studies on medical device prioritization. As little prior evaluative literature on this topic exists and as heterogeneous priority setting criteria are suggested to be equally legitimate [31], we are reluctant to quality appraise studies we include in the review or limit inclusion to only one type of study which may advance a particular prioritization methodology. This may imply more laborious and complex data analysis and may furthermore undermine the validity of any findings. Reviewing the literature obtained from such diverse sources, however, is greatly beneficial for hypothesis generation as it allows for consideration of multiple viewpoints and identification of minutiae associated with medical device prioritization and procurement for LMICs. In particular, it will allow for the mapping of all the different types of literature and potential methodological differences on this topic.

Second, we make no concerted effort to identify or include national policy documents relating to medical devices in this review. This is because the focus of the review is normative and concerns itself with the identification of procurement and prioritization methodologies within internationally disseminated recommendations and guidelines as well as research studies. We are thus interested in answering the question of how procurement and prioritization *should* take place considering current research and guideline materials. We acknowledge that national policies may in fact employ different procurement or prioritization methodologies, which we may fail to identify here and thus, encourage further inquiries into both the policy literature as well as the empirical implementation literature beyond. Should materials meet inclusion criteria, they will be selected for review. It is beyond the scope of this review to undertake an appraisal of all internationally available policy documents. Indeed, we caution that a systematic review of policies alone may fail to identify macro-level issues and themes relating primarily to international decision making paradigms (e.g. paradigms of international donor organizations or funding bodies supporting LMIC procurement). An inquiry into the normative bases of medical device procurement for LMICs is valuable in the initial exploration, and identification of issues, paradigms and processes is to be considered by decision makers. Review findings may provide a starting point to future policy analyses or research endeavours within this field.

Furthermore, the review may be limited in scope, as it is not designed to identify and include prioritization methodologies referring to entire intervention packages rather than devices or equipment. To make sure that applicable methodologies are not discounted, reviewers will consult experts in international health to identify any such relevant methodologies and discuss the findings of the current review in light of these.

An accurate understanding of medical device procurement and prioritization methods is of particular importance in resource-constrained settings with limited financial capabilities, human resource skills and health infrastructure. The findings of this systematic review will provide initial hypotheses as to what factors and stakeholders affect these processes and may aid in the formulation of a quality assurance framework able to provide LMIC decision makers with a rounded conceptualization of the topic.

## Abbreviations

HICs: high income countries; HIV: human immunodeficiency virus; LMICs: low- and middle-income countries (World Bank definitions apply); MSH: Management Sciences for Health; PRISMA: preferred reporting items for systematic reviews and meta-analyses; PROSPERO: international prospective register of systematic reviews maintained by the Centre for Reviews and Dissemination, University of York, UK; TB: tuberculosis; UNAIDS: the joint United Nations Programme in HIV/AIDS; UNFPA: the United Nations Population Fund; USAID: United States Agency for International Development; WHO: World Health Organization.

## Competing interests

The authors declare that they have no competing interests.

## Authors’ contributions

KD developed and drafted the current study protocol. YFC, SMH, CC and RL participated in the critical drafting of the protocol and offered advice on the research methodologies employed. All authors read and approved the final manuscript.

## Authors’ information

Karin Diaconu is a doctoral researcher exploring medical device prioritization and the use of health economic methodologies for prioritization purposes in low- and middle-income countries at the University of Birmingham. Yen-Fu Chen is a senior research fellow with considerable experience in health technology assessment and systematic reviews within the Warwick Centre for Applied Health Research and Delivery at the University of Warwick. Semira Manaseki-Holland is a senior clinical lecturer with substantial health service management experience in low- and middle-income settings at the University of Birmingham. Carole Cummins is a senior lecturer and researcher involved in the NIHR Birmingham and Black Country Collaboration for Leadership in Applied Health Research and Care (BBC-CLAHRC) at the University of Birmingham. Professor for Clinical Epidemiology, Richard Lilford is the director of the BBC-CLAHRC at the University of Birmingham and head of the Warwick Centre for Applied Health Research and Delivery at the University of Warwick.

## Supplementary Material

Additional file 1 **Definitions: Medical device procurement.** Definitions for medical device/equipment procurement and prioritization within procurement cycles are provided. Click here for file
