# Supplementary material for: Medical device procurement in low- and middle-income settings: protocol for a systematic review
Source: Syst Rev. 2014 Oct 21;3:118. doi: 10.1186/2046-4053-3-118 (PMC4211929; doi:10.1186/2046-4053-3-118)
Supplement: Additional file 1 — Definitions: Medical device procurement. Definitions for medical device/equipment procurement and prioritization within procurement cycles are provided. [file 2046-4053-3-118-S1.pdf]

Box 1: Definitions: Medical Device Procurement

The procurement of medical devices ranges from pre-purchase planning and identification of products to be purchased, to tendering for acquisitions and delivery of products.

Technology use, maintenance and decommissioning may impact upon future procurement practices through procedural evaluations of technology uptake, quality control or assurance procedures, as well as equipment field tests. Reflections on how equipment fares in deployment settings and its usability therein may thus impact upon future procurement.

Prioritization in procurement
Under resource and financing constraints, decision makers are forced to prioritize medical devices and equipment for purchasing and investment. This involves allocation of resources between competing medical equipment or devices and may result in the rank-ordering potential purchases according to explicitly stated or implicitly followed criteria. Considering the severe constraints faced by low- and middle-income country decision makers, prioritization and the criteria used within this process are of particular importance to decision makers, researchers and manufacturers.
